# A silent pheochromocytoma with regional metastasis and negative germline testing

**DOI:** 10.1530/EDM-25-0124

**Published:** 2026-03-26

**Authors:** Vasu Bansal, Sallam Alrosan, Sobrina S Mohammed, Hana Anan Hamdan, Shourya Tadisina

**Affiliations:** ^1^ Department of Internal Medicine, University of Missouri–Kansas City, Kansas City, Missouri, USA; ^2^ Department of Pathology, University of Missouri–Kansas City, Kansas City, Missouri, USA; ^3^ Department of Endocrinology, University of Missouri–Kansas City, Kansas City, Missouri, USA

**Keywords:** pheochromocytoma, paraganglioma, germline testing, metastasis, adrenal tumor

## Abstract

**Summary:**

Pheochromocytomas (PCCs) and paragangliomas (PGLs) are rare neuroendocrine tumors characterized by a universal potential for malignancy regardless of size or biochemical profile. While classic presentations involve paroxysmal hypertension, headaches, palpitations, and diaphoresis, an increasing proportion are detected incidentally through unrelated imaging. These ‘silent tumors’ often lack catecholamine-related symptoms and demonstrate absent or borderline biochemical abnormalities, signifying a substantial diagnostic challenge. Their indolent clinical profile may obscure malignant potential, leading to delayed intervention and missed opportunities for curative resection. We report a clinically and biochemically silent pheochromocytoma with regional lymph node metastasis, negative germline testing, and high-risk histopathologic features despite small tumor size and long-standing radiographic stability. This case illustrates that radiologic suspicion should prompt timely surgical management with appropriate perioperative blockade even in the absence of unequivocal biochemical confirmation. Lifelong surveillance remains essential with high-risk histology, irrespective of presentation.

**Learning points:**

- Biochemical silence does not imply benign pathology. Pheochromocytomas without catecholamine excess can still exhibit malignant potential, thereby highlighting the need for comprehensive testing in all cases.
- Discordant testing requires integrative assessment. Equivocal biochemical results should not preclude further evaluation or empiric preoperative blockade, especially when imaging features or clinical context raises suspicion.
- Tumor size is an unreliable risk surrogate. Even small adrenal lesions may harbor aggressive histopathology; risk stratification must incorporate pathology and molecular profiling.
- Genetic testing is indispensable but not definitive. Comprehensive germline testing informs prognosis and surveillance, yet negative results do not eliminate the possibility of malignant behavior.
- Long-term surveillance remains essential. High-risk features mandate sustained biochemical and imaging follow-up, regardless of tumor size, biochemical phenotype, or genetic background.

## Background

Pheochromocytomas (PCCs) and paragangliomas (PGLs) are rare neuroendocrine tumors arising from chromaffin tissue, with PCCs located in the adrenal medulla and PGLs found extra-adrenally (1). The World Health Organization (WHO) recommends describing these tumors as localized or metastatic, reflecting their universal potential for malignancy; 10–25% may eventually metastasize (2, 3, 4).

With the increased use of imaging for unrelated or surveillance purposes, incidental detection of PPGLs is becoming more common (5). This has led to the wider use of the term ‘silent’ PPGLs, referring to tumors that lack symptoms of catecholamine excess, though this terminology remains inconsistently defined (6).

## Case description

**Figure 1:**
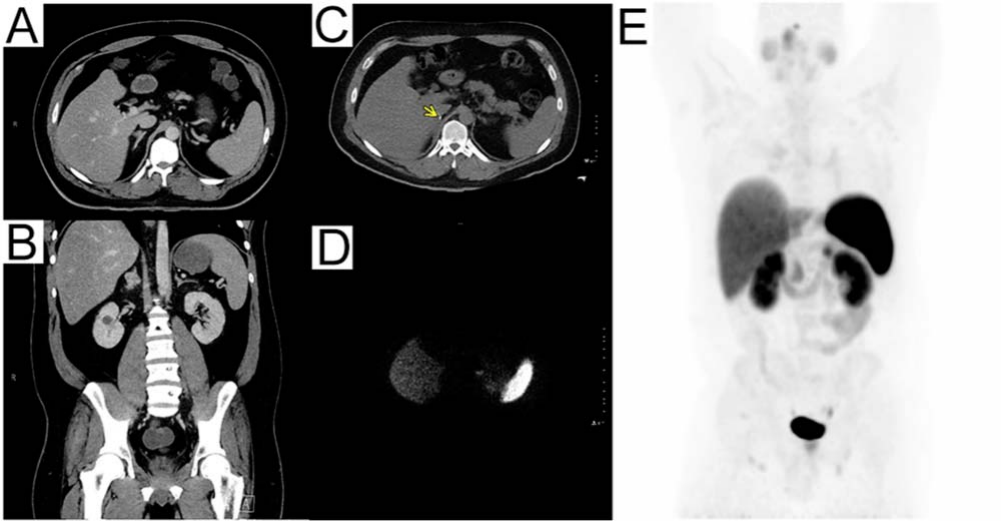
(A) Axial contrast-enhanced CT image demonstrates a 2.8 cm right adrenal mass with irregular contours and a heterogeneous enhancement pattern. (B) Coronal CT image further highlights the heterogeneity of the adrenal mass and reveals multiple sub-centimeter renal cysts. (C) Axial postoperative CT image shows postsurgical changes in the right adrenal bed; the arrow denotes a surgical clip in the adrenal fossa with no evidence of recurrent mass. (D) The corresponding axial ^68^Ga-DOTATATE PET image at the same level demonstrates no abnormal somatostatin receptor-avid uptake in the adrenal bed. (E) Whole-body ^68^Ga-DOTATATE PET maximum intensity projection (MIP) image shows no evidence of residual or metastatic disease.

A 38-year-old male with obesity (BMI: 32 kg/m^2^), chronic hepatitis C on treatment, and a previous history of intravenous drug abuse, quit over five years ago, was referred for evaluation of an incidentally discovered right adrenal mass. During hepatologic surveillance in 2024, a CT scan revealed a 2.7 × 2.4 cm right adrenal mass with irregular borders and non-contrast attenuation of 35 Hounsfield units. A dedicated adrenal CT obtained shortly after referral demonstrated a heterogeneous 2.8 × 2.8 cm mass averaging 75.9 HU with indeterminate absolute and relative washout (Fig. 1A and B). An adrenal MRI showed marked T2 hyperintensity, avid post-contrast enhancement, and absence of out-of-phase signal dropout. A review of outside imaging uncovered a 2019 CT demonstrating a 4 cm heterogeneous right-adrenal nodule that had never been investigated, establishing at least six years of radiographic persistence.

## Investigation

The patient remained normotensive and asymptomatic, denying headaches, palpitations, diaphoresis, flushing, weight loss, or hyperglycemia. Biochemical evaluation revealed a mildly elevated 24-h urinary normetanephrine level of 740 μg (reference upper limit: ∼400 μg/24 h), representing less than a twofold increase, with normal urinary metanephrine at 140 μg/24 h (ULN: ∼300 μg/24 h). Plasma fractionated metanephrines were within normal limits. The rest of the biochemical workup was negative. He denied use of any illicit drugs or medications that can cause false-positive urinary metanephrine elevation.

## Treatment

Given the discordance between the equivocal biochemical findings and the indeterminate imaging features, a decision was made to initiate empiric alpha-adrenergic blockade with low-dose doxazosin, which was titrated over two weeks. β-blockade was also initiated a week prior to surgery, and he underwent laparoscopic right adrenalectomy.

**Figure 2:**
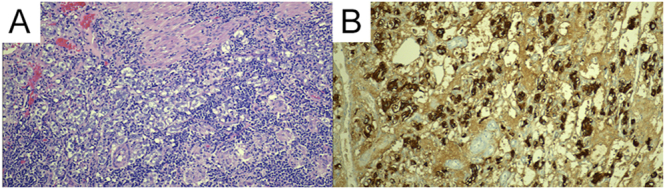
(A) Histopathology of the pheochromocytoma demonstrates a vaguely nested (zellballen) architecture, with notable cellular crowding and vesicular, overlapping nuclei. (B) Immunohistochemistry for Melan-A reveals diffuse cytoplasmic positivity within the tumor cells.

Surgery proceeded uneventfully under invasive hemodynamic monitoring; no catecholamine surges occurred. Gross examination revealed an encapsulated 2.9 cm tumor. Histology confirmed pheochromocytoma with partial capsular penetration and focal direct invasion of a pericapsular lymph node, corresponding to stage pT1 pN1. The Pheochromocytoma of the Adrenal gland Scaled Score (PASS) was 9/20, driven by diffuse growth, high cellularity, cellular spindling, pleomorphism, capsular invasion, and hyperchromasia; Ki-67 proliferation index was 5% (Fig. 2). Immunohistochemistry was positive for chromogranin, synaptophysin, vimentin, and GATA3 and negative for S-100, Melan-A, and HMB-45. Comprehensive germline testing (RET, VHL, SDHx, MAX, TMEM127, NF1, and FH) was negative, classifying the tumor as sporadic.

## Outcome and follow-up

Postoperative ^64^Cu-DOTATATE PET/CT showed no residual adrenal bed activity or distant radiotracer uptake; faint bilateral pulmonary foci were interpreted as probable infectious infiltrates, with interval chest imaging planned (Fig. 1C, D, E). ^64^Cu-DOTATATE PET/CT was chosen as the initial functional imaging modality due to its higher sensitivity for detecting metastatic pheochromocytoma and paraganglioma compared with ^123^I-metaiodobenzylguanidine (MIBG) scintigraphy. As no distant metastatic disease was identified, MIBG imaging was not pursued, given its primary role in evaluating eligibility for radiopharmaceutical therapy in disseminated disease.

The surveillance strategy includes plasma or urinary metanephrines every three months for the first year, semi-annually for the next two years, and then annually thereafter, complemented by periodic cross-sectional imaging. Antihypertensive therapy was discontinued immediately postoperatively, and his blood pressure remained stable with normal plasma metanephrines.

## Discussion

This case exemplifies the diagnostic complexity of clinically and biochemically silent pheochromocytoma. In our case, the 2.9 cm adrenal mass was discovered incidentally with no catecholamine-associated symptoms. Among available risk stratification tools, only the clinical feature-based PPGL probability score has been developed to estimate the likelihood of pheochromocytoma or paraganglioma based on presenting signs and symptoms at the time of biochemical evaluation. In contrast, other commonly cited scoring systems, including PASS, GAPP, and COPPS, are histopathology-based tools intended to assess malignant potential or metastatic risk after tumor resection rather than pre-test clinical probability. In our case the GAPP score based on pathology and available biochemical markers is 7 (large nests (+1), high cellularity (+2), adrenal capsular invasion (+1), Ki-67 5% (+2), and noradrenergic (+1)) which is consistent with a poorly differentiated tumor. Biochemical workup showed mildly elevated 24-h urinary normetanephrines but normal plasma fractionated metanephrines, findings that typically lower the clinical suspicion for pheochromocytoma. However, the atypical imaging features, including T2 hyperintensity and radiographic persistence over six years, warranted further investigation. Dopamine-secreting pheochromocytomas and paragangliomas represent an important diagnostic consideration in clinically and biochemically silent tumors with malignant potential. In this case, plasma dopamine and 3-methoxytyramine levels were not assessed preoperatively, which we acknowledge as a limitation, and chromogranin A levels were also not obtained.

However, the perioperative risk in pheochromocytoma is primarily determined by functional catecholamine activity. Accordingly, empiric α-adrenergic blockade followed by β-blockade was initiated prior to surgery, thereby reducing the risk of intraoperative catecholamine-mediated hemodynamic instability, including in the setting of potential dopamine-predominant secretion. As such, the absence of these additional biochemical measurements was unlikely to have altered perioperative management in this case.

Pheochromocytomas can be clinically silent or present with nonspecific symptoms, contributing to the complexity and nuance of diagnosis (7). They may arise through several complementary mechanisms. Increasing use of cross-sectional imaging and surveillance of at-risk patients has led to earlier detection, when tumor size and catecholamine output may be insufficient to produce hypertension or the classic symptom triad. In addition, the biochemical phenotype depends on tumor enzyme expression, with reduced phenylethanolamine N-methyltransferase (PNMT) favoring a predominantly noradrenergic pattern and diminished dopamine-β-hydroxylase resulting in dopamine-predominant secretion. Because dopamine has limited α- and β-adrenergic activity and may exert vasodepressor effects, dopamine-producing tumors may remain normotensive or present without typical adrenergic symptoms.

The pattern of catecholamine release further influences clinical presentation, as sustained low-level secretion may be better tolerated than episodic surges and may be further attenuated by physiological adaptation, including intravascular volume changes and adrenoceptor desensitization. Finally, even when circulating norepinephrine is elevated, hormonal secretion from a tumor may produce less pronounced hemodynamic effects than sympathetic neuronal activation, contributing to an apparently silent presentation (6).

Given the potential for underdiagnosis in such presentations, this case supports a low threshold for empiric alpha- and beta-blockade when radiologic concern is high, even in the absence of unequivocal laboratory evidence.

Surgical resection revealed histopathologic features associated with malignant potential, including partial capsular invasion, regional lymph node involvement (pN1), a PASS of 9, and a Ki-67 index of 5%. Histological assessment remains crucial in prognostication and management, particularly when biochemical markers are inconclusive (8). While metastasis remains the only definitive marker of malignancy per WHO criteria (2), this case confirmed regional metastatic disease. Notably, a recent systematic review of silent pheochromocytomas and paragangliomas reported metastases in approximately 25% of cases, underscoring that silent does not mean benign (6).

Importantly, the tumor’s small size did not preclude malignant behavior, challenging the assumption that biologic aggressiveness correlates with tumor diameter. This case reinforces that tumor size alone is not predictive of clinical course and high-risk features may still be present in biochemically indolent tumors.

Moreover, most metastatic pheochromocytomas are associated with germline mutations, particularly in SDHB and other cluster 1 susceptibility genes (9). However, comprehensive genetic testing in this case was negative, making it a rare sporadic presentation of aggressive disease. This reinforces the importance of genetic testing in all PPGL cases, regardless of biochemical phenotype or family history, to guide risk stratification and follow-up. However, several caveats must be acknowledged. As with any clinical test, there is a small but real false-negative error rate. In addition, not all susceptibility genes for PPGLs have been identified. Some mutation types, such as large deletions or duplications, may require specific techniques beyond standard sequencing, which are not always included in routine panels. While most laboratories apply stringent quality controls to minimize false positives, such as sequencing both DNA strands and using duplicate samples, false negatives are harder to eliminate, particularly for complex structural variants or poorly covered genomic regions.

Furthermore, we do not have information on possible somatic mutations in this case, which may also contribute to tumor behavior and malignant potential. Thus, negative germline testing does not rule out underlying molecular drivers of disease, and long-term surveillance remains essential, especially when histopathology reveals high-risk features.

Finally, the surveillance strategy for this patient, including serial plasma or urinary metanephrines and periodic imaging, aligns with current guidelines and reflects the lifelong risk of recurrence, particularly in tumors with high-risk histologic features.

## Conclusion

This case highlights the diagnostic and prognostic challenges posed by small, clinically silent pheochromocytomas with inconclusive biochemical profiles. Despite its modest size and absence of symptoms, the tumor demonstrated aggressive histopathologic features and regional metastasis, underscoring that tumor size and biochemical indolence do not preclude malignant potential. Negative germline testing supported a sporadic etiology, but the absence of somatic mutation data leaves unanswered questions about additional molecular drivers. Fortunately, an increasing number of pheochromocytomas and paragangliomas are now identified prior to the onset of symptoms, either incidentally through imaging or via targeted screening in genetically at-risk individuals. This case underscores the need for continued clinical vigilance and thorough evaluation of adrenal lesions, regardless of symptomatology or initial biochemical impression.

## Declaration of interest

The authors declare that there is no conflict of interest that could be perceived as prejudicing the impartiality of this work.

## Funding

This work did not receive any specific grant from any funding agency in the public, commercial, or not-for-profit sector.

## Patient consent

Written informed consent for publication of their clinical details and/or clinical images was obtained from the patient.

## Author contribution statement

VB and SA wrote the original draft of the manuscript. HAH prepared the pathology images. SSM wrote, reviewed, and edited the manuscript. ST supervised the study and wrote, reviewed, and edited the manuscript. We confirm that all listed authors have reviewed and approved the revised manuscript and are in agreement with the current version of the article.
